# The role of South-North partnerships in promoting shared learning and knowledge transfer

**DOI:** 10.1186/s12992-017-0289-6

**Published:** 2017-08-22

**Authors:** Lopa Basu, Peter Pronovost, Nancy Edwards Molello, Shamsuzzoha B. Syed, Albert W. Wu

**Affiliations:** 1 World Health Organization Service Delivery & Safety Department, 750 East Pratt Street, 15th floor, Baltimore, MD 21202 USA; 2 Armstrong Institute for Patient Safety & Quality, 750 East Pratt Street, 15th floor, Baltimore, MD 21202 USA; 3 Armstrong Institute for Patient Safety & Quality; Johns Hopkins Medicine, 750 East Pratt Street, 15th floor, Baltimore, MD 21202 USA; 4 World Health Organization Service Delivery & Safety Department, 20, Avenue Appia, Rm 4176, CH-1211 Geneva, Switzerland

**Keywords:** Reverse innovation, Partnerships, Patient safety, Learning

## Abstract

While it is clear that hospitals in developing countries need to improve quality of health services and improve patient safety, hospitals in high resource countries need to do the same. Most often the focus on improvement through institutional health partnerships involves hospital teams from high resource settings attempting to aid and teach hospital staff in low resource settings, particularly in Africa. However these efforts to provide assistance may be more satisfying and sustainable if we understand that partnership learning is bi-directional whereby hospital teams from high resource settings also benefit. One particular partnership-based model that demonstrates this benefit to high resource partners is the World Health Organization African Partnerships for Patient Safety (APPS). Johns Hopkins Medicine Armstrong Institute for Patient Safety & Quality (AI) through the APPS model has co-created twinning partnerships with hospitals in Uganda, South Sudan & Liberia. This commentary aims to deconstruct specific learnings that have benefited the Johns Hopkins AI community through the APPS partnership.

## Background

Patient safety lessons too often focus on what high-income health systems have often done to inform and guide low-income health systems. However, there is growing evidence that practical and simple approaches to deliver people-centered care to improve the quality of healthcare elicited from low-income countries which have not been fully explored by high-income countries, a process called ‘reverse innovation’ [1, 2] needs further examination. Interest in this field is increasing in global health dialogues and building a growing global pool of knowledge [3] is a critical next step towards unpacking the specific opportunities for South-North learning.

## Global innovation flow

To fully realize the potential for effective flow of innovations, healthcare providers from low and high-income countries need to create trusting, mutually respectful relationships and develop structures and processes to support peer learning. The World Health Organization (WHO) is doing this through its African Partnerships for Patient Safety (APPS) (http://www.who.int/patientsafety/implementation/apps/en/), a program that highlights the importance of human interaction through site visits, joint trainings, bi-directional learning and co-development of new innovations.

One example of an APPS partnership is between the Johns Hopkins Medicine/Armstrong Institute for Patient Safety (AI) and partnering hospitals in Liberia, Uganda & South Sudan. As part of the APPS program, teams have interacted in-person and virtually. During these exchanges, the teams co-created their purpose, core values and selected topics for shared learning.

People-centered practices in Africa have provided clear opportunities for learning even in renowned institutions of the globe. South-to-north lessons have also emerged on minimizing waste in healthcare and on building sustainable, environmentally conscious hospitals (Green Living). Below we summarize some of the key lessons we have learned.

## People-centered care

Enhancing the patient experience and delivering people-centered care is a goal for virtually every hospital on the planet. Academic institutions have researched, analyzed and collated distinct best practices in people-centered care as a means to discover tangible actions that providers and hospital leadership can perform to provide comprehensive people-centered care. High-income health systems are now extending visitation hours, conducting multidisciplinary rounds in patient’s rooms, creating primary care medical homes and many others [4]. Each of these is potentially helpful, yet all are rooted in a narrative that patients and families are separate and need to be reunited as part of the care team.

Our African colleagues taught us a different narrative. Patients, families and the care team are one; they were never separated and thus do not need to be reunited. Many public African hospitals do not have private rooms, thus visitation hours are generally open and rounds are always at the bedside. Due to resource challenges and in response to health workforce constraints, families and guardians often feed and bathe patients, innately providing a people-centered approach whereby patient families are actively engaged in patient care. The family works with the care team from the beginning to provide basic services for the patient.

In response to an overburdened work force, patient guardians, and ‘barefoot patient advocates’ are directly involved in the patient’s activities of daily living including bathing, feeding, cooking, transporting patients for test and even assisting with rehabilitation exercises [5]. These guardians are actively present and engaged with their hospitalized loved ones as many families live far from the hospital and often have to live in guardian shelters on hospital grounds. Although there are potential burdens on the family unit (such as lost work wages), the direct engagement of family members allows for increased trust for patients and bedside advocates to promote transparency in care.

Our African partners taught Johns Hopkins a new narrative of what people-centered care might look like. Johns Hopkins borrowed many of these ideas which include providing families a menu of tasks they can perform to help care for their loved ones and creating a health buddy, a family member or friend who the patient assigns to help them with their care.

## Choosing wisely

The U.S. is working to reduce healthcare costs. For example, the American Board of Internal Medicine Foundation (ABIMF) launched *Choosing Wisely* in 2012 with a goal of reducing wasteful or unnecessary medical tests, treatments and procedures. Many healthcare providers in high-income countries proudly focus on choosing wisely by promoting practicing practices that avoid unnecessary testing and procedures*.*

Our African partners are routinely forced to choose wisely out of necessity. Hospitals in low-income countries, doing much with little, avoiding waste, is the norm not the exception. Our African partners talked about doing surgery without pre-operative or post-operative laboratory tests or images and were forced to rely upon the clinical exam and patient’s history. In low-income settings all over the world, the concept of ‘doing much with little’ is not an innovation, but a survival mechanism. Providers and families have adapted by working together, being mindful of the task at hand, and being creative with a collective effort that involves communities and beyond. Our partners working in low-income countries often share system-wide resources – for example sharing local incinerators to dispose of medical waste – to help mediate regional needs of health facilities. A critical eye on the “resource-value” equation within care provision is thus a norm.

Our African partners have taught us a new narrative of what waste reduction looks like, making clearly apparent how wasteful healthcare is in our institution and how much we have to learn. Choosing wisely has felt small.

## Green living

Most organizations in high-income countries, including hospitals, are working to reduce their carbon emissions and become greener. Yet, few high-income hospital leaders are even aware of how much specific energy they consume and what energy costs them. A first step in any improvement effort for facilities to go green requires a knowledge of how they use utilities, medical supply/waste and other infrastructure resources that contribute to environmental degradation.

The Chief Medical Officer at Jackson F Doe hospital in Liberia can quote the daily kilowatts used by his hospital and how much money he spends on energy. Energy costs often consume up to half of a hospital’s budget, and as a result energy use and costs are on the performance dashboard of many low-income hospitals. Being conscious of how each dollar is spent on energy to power a hospital reveals how limited resources have forced senior level partners to be actively engaged with considering energy utilization.

Our African partners have taught us that our green efforts are grossly underdeveloped. If we are to get serious, high-income hospital leaders will need to routinely monitor and reduce their energy use and costs.

## Conclusion

Hospital twinning partnerships provide an active channel for the power of diverse ideas to transcend national borders. Such partnerships underline how much high-income hospitals can learn from low-income hospitals, if we are humble enough to listen and learn. One way to start might be to move from thinking of this process as “reverse innovation” to “global innovation flow” in recognition of true shared learning. As we must recognize, good ideas with impact – of course – emerge everywhere.

## References

[1] Immelt J, Govindarajan V, Trimble C (2009). How GE is disrupting itself. Harvard Bus Rev.

[2] Syed SB, Dadwal V, Martin G (2013). Reverse innovation in global health systems: towards global innovation flow. Glob Health.

[3] Reverse innovation in global health systems: learning from low-income countries. http://www.globalizationandhealth.com/series/reverse_innovations (accessed 10 Nov 2015).

[4] Berenson RA, Hammons T, Gans DN (2008). A house is not a home: keeping patients at the center of practice redesign. Health Aff (Millwood).

[5] Basu L, Frescas R, Kiwelu H (2014). Patient guardians as an instrument for person centered care. Glob Health.

